# Supplementary figures and images for: Differential Participation of Plant Ribosomal Proteins from the Small Ribosomal Subunit in Protein Translation under Stress
Source: Biomolecules. 2023 Jul 21;13(7):1160. doi: 10.3390/biom13071160 (PMC10377644; doi:10.3390/biom13071160)

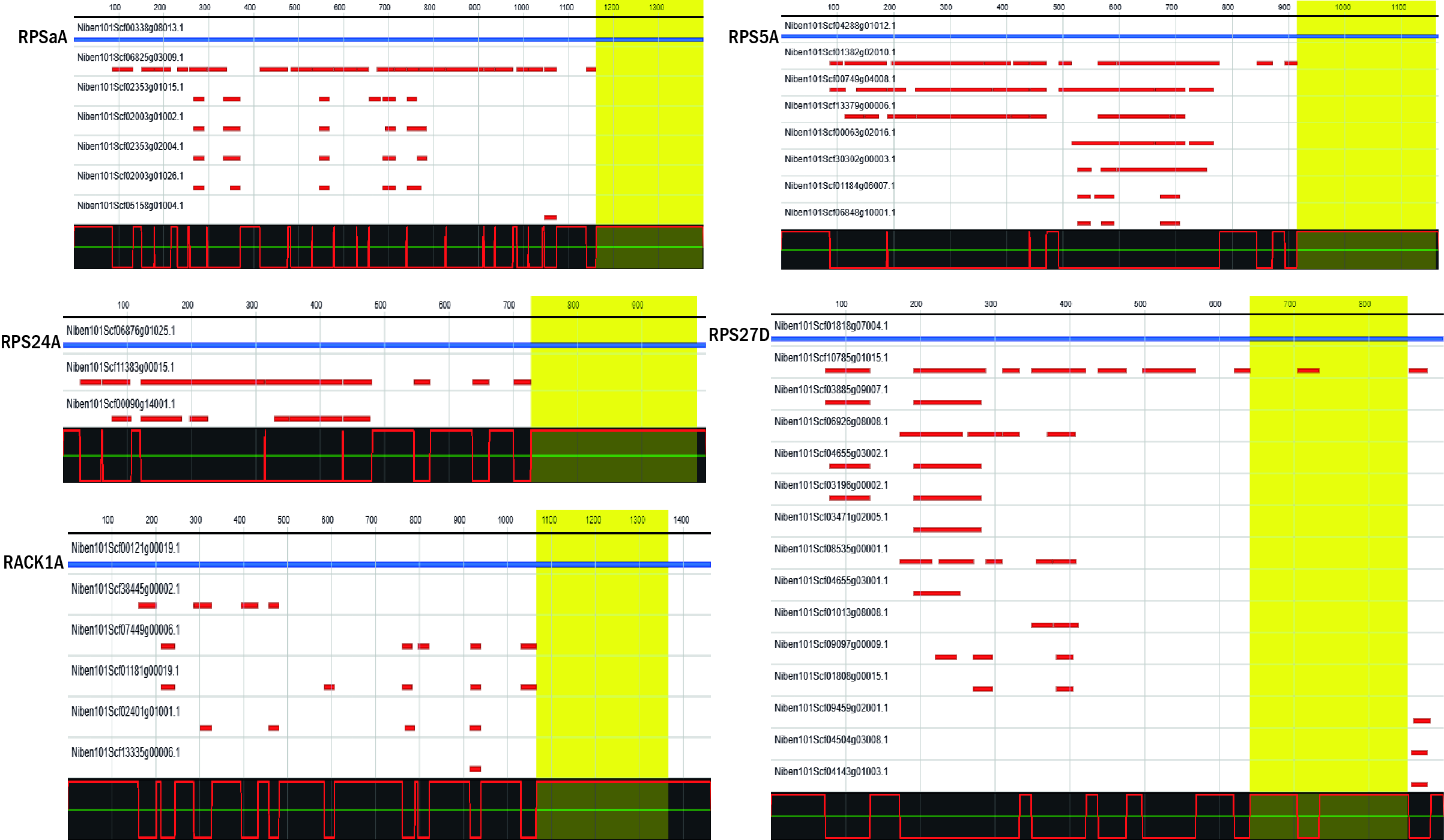

Supplement: Supplementary file 1 [file biomolecules-13-01160-s001.zip › Figure S1.tif]

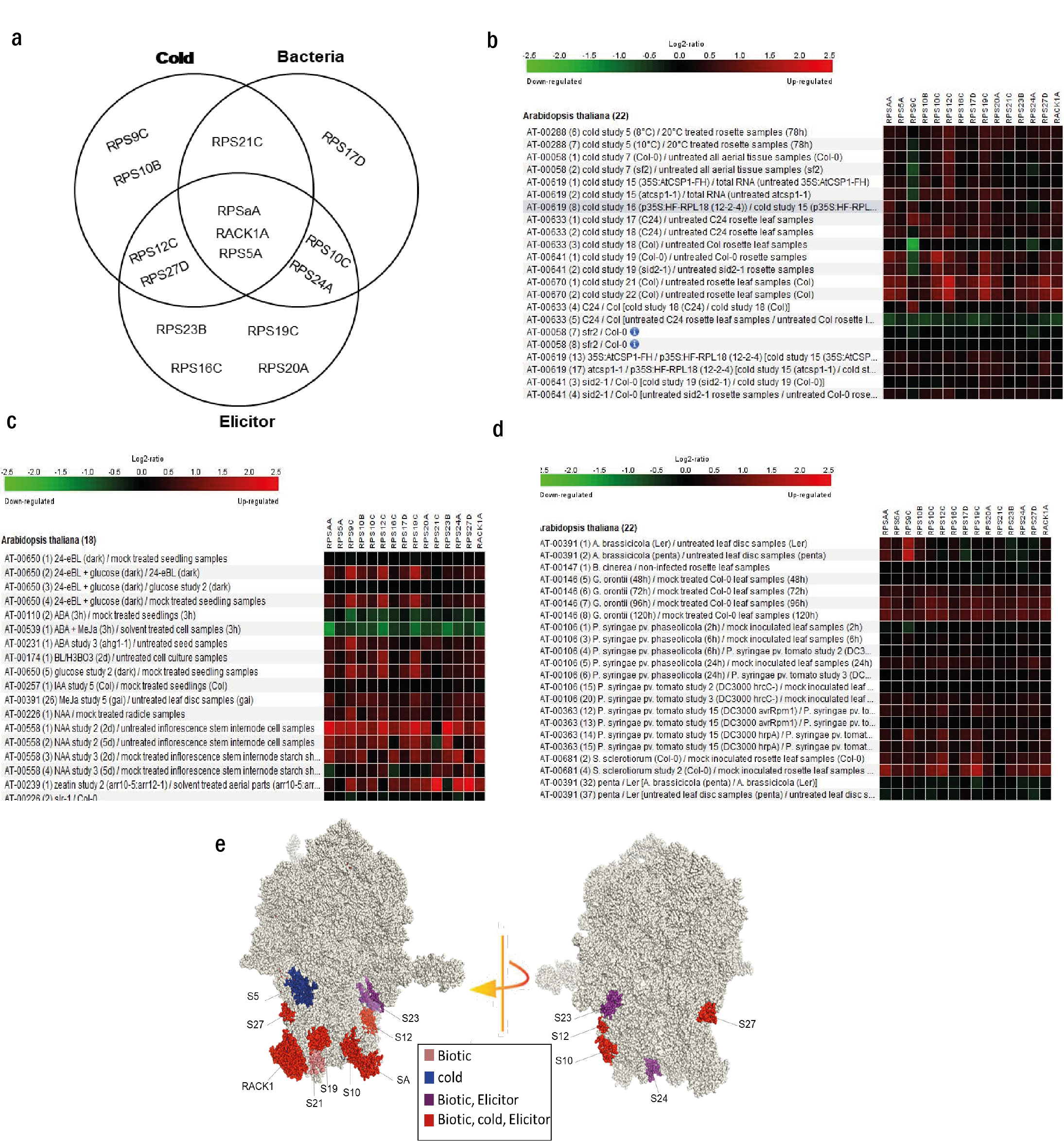

Supplement: Supplementary file 1 [file biomolecules-13-01160-s001.zip › Figure S2.tif]
